# Incidence of Avoidable 30-Day Readmissions Following Hospitalization for Community-Acquired Pneumonia in France

**DOI:** 10.1001/jamanetworkopen.2022.6574

**Published:** 2022-04-08

**Authors:** Bastien Boussat, Fabiana Cazzorla, Marion Le Marechal, Patricia Pavese, Anne-Laure Mounayar, Elodie Sellier, Jacques Gaillat, Boubou Camara, Bruno Degano, Mylène Maillet, Xavier Courtois, Magali Bouisse, Arnaud Seigneurin, Patrice François

**Affiliations:** 1 Service d’épidémiologie et évaluation médicale, CHU Grenoble-Alpes, Grenoble, France; 2 Laboratoire TIMC-IMAG, UMR 5525 Joint Research Unit, Centre National de Recherche Scientifique, Université Grenoble-Alpes, France; 3 O’Brien Institute for Public Health, University of Calgary, Calgary, Alberta, Canada; 4 Service des maladies infectieuses, CHU Grenoble-Alpes, Grenoble, France; 5 Service d’information médicale, CHU Grenoble-Alpes, Grenoble, France; 6 Service d’information et d’évaluation médicale, Centre hospitalier Annecy-Genevois, Épagny-Metz-Tessy, France; 7 Service de pneumologie, CHU Grenoble-Alpes, Grenoble, France; 8 Service des maladies infectieuses, Centre hospitalier Annecy-Genevois, Épagny-Metz-Tessy, France

## Abstract

**Importance:**

Rates of 30-day readmissions following hospitalization for pneumonia are used to publicly report on hospital performance and to set financial penalties for the worst-performing hospitals. However, the rate of avoidable readmission following hospitalization for pneumonia is undefined.

**Objective:**

To assess how often 30-day readmissions following hospitalization for community-acquired pneumonia (CAP) are avoidable.

**Design, Setting, and Participants:**

This cohort study analyzed the results of an independent review of readmissions following hospitalization for CAP within 30 days among patients discharged from 2 large hospitals in France in 2014. Structured clinical records including clinical information (ie, baseline characteristics, physical examination, laboratory findings, x-ray or computed tomography scan findings, discharge plan, and treatments) for both index and readmission stays were independently reviewed by 4 certified board physicians. All consecutive adult patients hospitalized in 2014 with a diagnosis of CAP in our 2 eligible hospitals were eligible. All analyses presented were performed in March 2021.

**Main Outcomes and Measures:**

Avoidable readmission within 30 days of discharge from index hospitalization. The likelihood that a readmission was avoidable was quantified using latent class analysis based on the independent reviews. A readmission was considered avoidable if Bayes posterior probability exceeded 50%.

**Results:**

The total analytical sample consisted of 1150 index hospital stays with a diagnosis of CAP, which included 651 (56.6%) male patients. The median (IQR) age for all patients was 77.8 (IQR, 62.7-86.4) years. Out of the 1150 index hospital stays, 98 patients (8.5%) died in hospital, and 108 (9.4%) unplanned readmissions were found. Overall, 15 readmissions had a posterior probability of avoidability exceeding 0.50 (13.9% of the 108 unplanned readmissions; 95% CI, 8.0%-21.9%). The median (IQR) delay between the hospital discharge index and readmission was considerably shorter when readmission was deemed avoidable (4 [6-21] days vs 12 [2-18] days; *P* = .02).

**Conclusions and Relevance:**

Only a small number of readmissions following hospitalization for CAP were deemed avoidable, comprising less than 10% of all readmissions. Shorter time interval between hospitalization discharge and readmission was associated with avoidability.

## Key Points

**Question:**

How many 30-day readmissions following hospitalization for community-acquired pneumonia are avoidable?

**Findings:**

In a cohort study including 1150 index hospital stays with a diagnosis of community-acquired pneumonia, multiple physician review analyzed by latent class analysis found only 15 readmissions were avoidable (13.9% of the 108 unplanned readmissions).

**Meaning:**

The low rate of avoidable readmissions found in this study contradicts the logic of the Hospital Readmission Reduction Program, which is based upon the notion that they should be mostly avoidable.

## Teaser

This cohort study of French patients with community-acquired pneumonia examines the prevalence of avoidable hospital readmission through a statistical analysis of manual physician case review.

## Introduction

In the US, the Centers for Medicare & Medicaid Services (CMS) began using pneumonia readmission as a core metric in pay-for-performance programs on October 1, 2012.^1^ In the Hospital Readmission Reduction Program (HRRP), hospitals with higher than expected 30-day readmission rates for patients with several target conditions, including pneumonia, have their CMS payments reduced.^2^ The underlying logic of the HRRP is based upon the notion that short-term readmission is often an avoidable adverse outcome reflecting suboptimal quality of care during index hospitalization. Numerous flaws have been reported, including that deaths without hospitalization favorably affect the HRRP metric, and that the prediction model uses only hospital claims data with imperfect validity.^3,4^ In Europe, England, Germany, Denmark, and France have introduced pay-for-performance programs that include 30-day readmission rates as a core metric and are therefore exposed to similar flaws.^5,6^

Shorr et al^7^ reported other flaws concerning pneumonia readmission. Given the complex specificity of pneumonia, some hospital readmissions are unavoidable. For example, certain pathogens have been shown to be associated with higher rates of rehospitalization.^8^ More generally, the HRRP readmission model cannot adequately account for medical complexity, as many pieces of information are missing from hospital claims databases. The rate of readmissions deemed avoidable is an open question, especially if it must be high enough for readmission to be considered as a reliable quality-of-care measure.^9^

Solid evidence about the true rates and determinants of avoidable pneumonia readmissions are lacking in prior studies. Several models have sought out correlation with baseline characteristics but have not been able to precisely identify the circumstances of readmission that could be deemed avoidable.^10,11^ Moreover, most of the studies aimed at assessing avoidability have not used recommended guidelines and have reported heterogeneous results.^12,13,14,15,16^ Subjectivity always exists when determining the extent to which readmissions are avoidable, despite guidelines intended to minimize error. To counteract this, the parameters required for reviewing readmissions must include precise clinical information, and multiple reviewers are essential when dealing with avoidability.^17^

Through a multicenter cohort study of hospitalized patients diagnosed with community-acquired pneumonia (CAP), we aimed to identify the proportion of pneumonia readmissions that might justifiably be deemed avoidable. We performed multiple reviews and, using latent class models, calculated the likelihood that readmission of a given patient was indeed avoidable. In addition, we carried out an analysis of the potential factors associated with avoidability. We hypothesized that a significant proportion of readmissions were nonavoidable.

## Methods

### Study Design and Settings

This retrospective, observational cohort study was conducted at a university-affiliated hospital and a general hospital in France. The first, the Grenoble University Hospital, has 1362 acute care beds. The second is Annecy General Hospital, with 660 acute care beds. The rationale, methods, and study sample have been reported in detail elsewhere.^18^ The protocol for this study was approved by the Comité de Protection des Personnes Sud-Est V. The consent for data collection through medical record review and the use of corresponding administrative discharge data was sought under a regime of nonopposition (ie, patient opt-out): after appropriate written information is delivered by regular mail, data were collected except in case of opposition from the patient. This study followed the Strengthening the Reporting of Observational Studies in Epidemiology (STROBE) reporting guideline.

### Population

We included all consecutive adult patients aged 18 years or older hospitalized in 2014 in either hospital with a CAP diagnosis. Eligible stays included adult patients (ie, over age 18 years) with an *International Statistical Classification of Diseases and Related Health Problems, Tenth Revision *(*ICD-10*) code of main diagnosis, related diagnosis, or significant associated diagnosis of low acute respiratory infection (eTable 1 in the Supplement).

After data entry, we excluded cases of lower respiratory infection without CAP in accordance with the following diagnostic criteria: diagnosis clearly mentioned in the hospital report; patient record contained a notation of 1 or more respiratory symptoms (cough, sputum production, dyspnea, tachypnea, or pleuritic pain), 1 or more auscultation findings (rale or crepitation), and 1 or more signs of infection (temperature above 38 °C, shivering, or white blood cell count below 4 or above 10 Giga/L), and a new pulmonary infiltrate on chest x-ray or computed tomography (CT) scan. For each included stay, the first all-cause readmission within 1 year of discharge was included, regardless of the main, related, or significant associated diagnosis.

### Data Collection

Data sources included patient electronic medical records and hospital discharge administrative data. To ensure optimal quality, 2 clinical research assistants entered all data collected electronically using a structured clinical record and a protected web-based data collection system. The clinical research assistants had received formal training in the methods of data abstraction and recording.

The following variables were recorded for each index hospitalization: patient and hospital stay identifiers; baseline patient characteristics, including demographics, preexisting comorbid conditions, pneumonia severity index risk class, physical examination and laboratory findings on admission, x-ray or CT scan findings within 48 hours of admission, and initial microbiological work-up; in-hospital antibiotic therapy and associated treatments; index hospital admission course (intensive care unit [ICU] admission, pneumonia-related and unrelated complications); physical examination and laboratory findings at discharge; and discharge plan and treatments.

Furthermore, the following variables were recorded for the first all-cause readmission within 1 year of discharge: patient and hospital stay identifiers; physical examination and laboratory findings on readmission, x-ray or CT scan findings within 48 hours of readmission; ICU admission; pneumonia-related and unrelated complications; and primary and secondary reasons for readmissions.

### Physician Reviews

Data on hospital stays with confirmed CAP with readmission within 30 days of discharge were submitted to 4 medical experts to evaluate the unplanned nature of readmission, its avoidable nature, and the causes of readmissions. We recruited a convenience sample of 9 board-certified physicians with experience in managing CAP, including 3 infectious disease specialists, 3 pulmonologists, and 3 clinical epidemiology specialists. Four panelists reviewed all readmission cases, including at least 1 infectious disease specialist, 1 pulmonologist, and 1 clinical epidemiologist (ie, the fourth panelist was either an infectious disease specialist, a pulmonologist or an epidemiologist). Cases were randomly assigned to each expert. To prevent evaluation bias, a clinical research assistant checked discharge letters and prescriptions to identify the physician in charge of the patient’s care. If one of the randomly selected reviewers was identified as in charge for the hospitalization, another reviewer from the same specialty replaced him or her. The panelists were requested to independently review medical records for both index hospitalization and readmission.

For nature of readmission, reviewers assessed the planned or unplanned nature of the readmission. When the 4 experts disagreed, a fifth expert was asked to decide on the planned or unplanned nature of the readmission. Investigation for planned readmissions did not go further. Consistent with van Walraven et al,^9^ reviewers used a 6-point ordinal scale to determine whether the readmission was an adverse event and whether the readmission could have been avoided (eTable 2 in the Supplement). A readmission with a rating above 3 in both domains was classified as avoidable. The panelists also assigned the primary reason for each readmission (regardless of avoidability) using 11 mutually exclusive categories^19^: unforeseen readmission for a new affection, complication of surgical care, complication of nonsurgical care, drug-related adverse event, premature discharge, discharge with a missing or erroneous diagnosis or therapy, other type of inadequate discharge, failure of postdischarge follow-up care, inadequate patient behavior, relapse or aggravation of a previously known condition, and social readmission.

### Outcome Measure

The primary outcome measure was avoidable readmission within 30 days of discharge from index hospitalization. The likelihood that a readmission was avoidable was quantified using latent class analysis based on the independent reviews by 4 panelists. A readmission was considered avoidable if Bayes posterior probability exceeded 0.50.^9^

### Statistical Analysis

We used descriptive statistics for reporting continuous (mean or median) and categorical (numbers and percentages) variables. We performed latent class analysis to quantify the probability that a readmission was avoidable, based on the independent classification by 4 panelists. The same approach was previously used by others.^9^ Briefly, latent class analysis is a statistical approach that assigns individuals to 2 or more latent classes (in our case, true avoidability) based on a set of observed categorical variables (in our case, avoidability rated by multiple reviews). The latent variable cannot be observed directly; instead, it is measured indirectly by using multiple observed variables.

We specified a 2-class model that reflects the dichotomy of avoidable vs unavoidable readmission. The independent classification of readmission conducted by each of the 4 experts was entered as observed categorical variables. We derived from the latent class model a Bayes posterior probability of avoidability for each individual case of readmission. The model-based sensitivity, specificity, and Youden index of each expert’s classification of readmissions as avoidable was calculated.

Patient-stay characteristics were compared between avoidable and nonavoidable readmissions using the χ^2^ test or Fisher exact test where appropriate for categorical variables and the *t* test or nonparametric Wilcoxon test for continuous variables. A 2-sided *P* < .05 was considered statistically significant. All statistical analyses were performed in 2021 using Stata Standard Edition version 16 (StataCorp).

## Results

### Patient Characteristics

From January 1, 2014, to December 31, 2014, 1523 hospital stays with an *ICD-10* diagnosis code of pneumonia were identified (Figure). After removing 186 hospital stays based on our exclusion criteria and 187 hospital stays with a diagnosis other than CAP, our analytical sample consisted of 1150 index hospital stays. The median (IQR) age of these patients was 77.8 (IQR 62.7-86.4) years, and 651 patients (56.6%) were men. Overall, 98 patients (8.5%) died in hospital and 184 were readmitted within 30 days of discharge, representing an early readmission rate of 17.5% (184 of 1052 patients; 95% CI, 15.2%-19.9%). Out of these 184 stays, 108 were classified by the panelists as unplanned and were included in the avoidability analysis (10.3% of discharges; 95% CI, 8.6%-12.3%; 58.7% of readmissions; 95% CI, 51.5%-65.6%).

**Figure.**
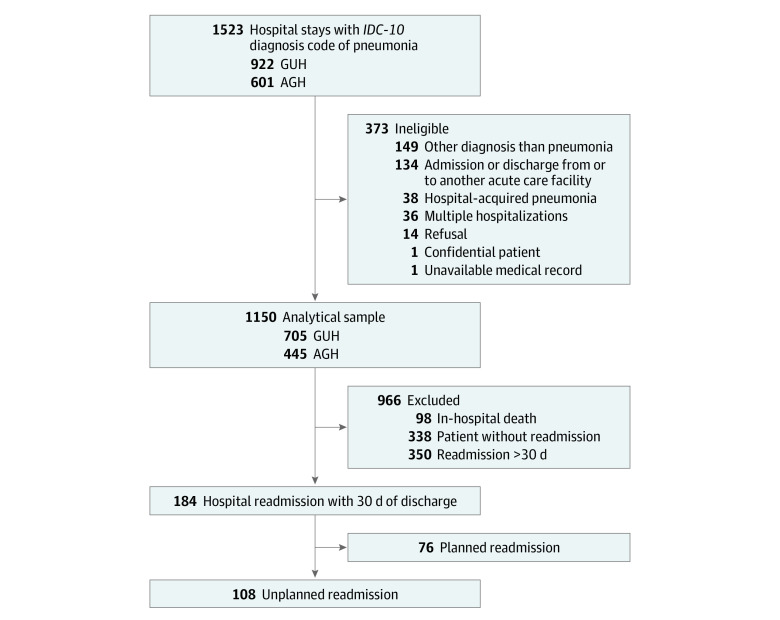
Flowchart of Study Population AGH indicates Annecy General Hospital; GUH, Grenoble University Hospital; *ICD-10*, *International Statistical Classification of Diseases and Related Health Problems, Tenth Revision*.

Among the 108 unplanned readmissions, the patient median age was 77.8 (65-86) years, 44 (40.7%) were women and 16 (15.0%) were nursing home residents. Median length of stay of index hospitalization was 9 (6-14) days (Table 1). A vast majority of the population had high-risk classes (IV-V) in the pneumonia severity index (72 patients [66.7%]).

**Table 1.** Population Characteristics

Characteristics	Patients, No. (%)	
	Total (n = 1150)	Unplanned readmission (n = 108)
Sex		
Men	651 (56.7)	64 (59.3)
Women	499 (43.3)	44 (40.7)
Age, median (IQR), y	77.8 (62.6-86.4)	77.8 (64.6-86.3)
Preadmission residence		
Private residence	940 (82.7)	88 (82.2)
Nursing home	169 (14.9)	16 (15.0)
Other	28 (2.5)	3 (2.8)
Length of stay, median (IQR), d	8 (4-13)	9 (6-14)
Medical history and comorbidities		
Neoplastic disease	140 (12.2)	17 (15.7)
Liver disease	49 (4.3)	8 (7.4)
Kidney disease	182 (15.8)	17 (15.7)
Cerebrovascular disease	164 (14.3)	19 (17.6)
Hemiplegia or paraplegia	46 (4.0)	5 (4.6)
Congestive heart failure	150 (13.0)	17 (15.7)
Prior myocardial infarction	103 (9.0)	8 (7.4)
Peripheral vascular disease	127 (11.0)	13 (12.0)
Diabetes	259 (22.5)	27 (25.0)
Chronic obstructive pulmonary disease	204 (17.7)	15 (13.9)
Rheumatologic disease	78 (6.8)	6 (5.6)
Peptic ulcer disease	60 (5.2)	7 (6.5)
Acquired Immunodeficiency Syndrome	8 (0.7)	2 (1.9)
Physical examination findings on admission^a^		
Altered mental status	230 (20.0)	26 (24.1)
Respiratory rate ≥30/min	180 (15.7)	17 (15.7)
Systolic blood pressure <90 mm Hg	107 (9.3)	10 (9.3)
Temperature <35 °C or ≥40 °C	28 (2.4)	1 (0.9)
Pulse rate ≥125/min	127 (11.0)	11 (10.2)
Laboratory and radiographic findings on admission^a^		
Arterial pH <7.35	57 (5.0)	4 (3.7)
Blood urea nitrogen ≥30.8 mg/dL	321 (27.9)	30 (27.8)
Sodium <130 mEq/L	58 (5.0)	3 (2.8)
Glucose ≥252.3 mg/dL	54 (4.7)	3 (2.8)
Hematocrit <30%	81 (7.0)	14 (13.0)
Partial pressure of arterial oxygen <60 mm Hg	212 (18.4)	15 (13.9)
Pleural effusion	204 (17.7)	22 (20.4)
Pneumonia severity index class IV-V	727 (63.2)	72 (66.7)
Charlson Comorbidity Index >0	876 (76.2)	90 (83.3)

SI conversion factors: To convert urea nitrogen to millimoles per liter, multiply by 0.357; sodium to millimoles per liter, multiply by 1.0; glucose to millimoles per liter, multiply by 0.0555; hematocrit to proportion of 1.0, multiply by 0.01; partial pressure of arterial oxygen to kilopascal, multiply by 0.133.

^a^ Missing values were considered as normal. Values were missing for preadmission residence (1 patient), respiratory rate (61 patients), pH (39 patients), blood urea nitrogen (1 patient), glucose (19 patients), and partial pressure of arterial oxygen (39 patients).

### Avoidable Readmissions

After physician review of the 108 patients with CAP, 15 readmissions had a posterior probability of avoidability exceeding 0.50 (13.9%; 95% CI, 8.0%-21.9%). The mean of the 108 posterior probabilities was 13.6% (95% CI, 7.9-19.2). This exact fit between the proportion of readmissions with posterior probabilities exceeding 0.50 and the mean validated the fit of the latent class model. Eighty-five of the 108 unscheduled readmissions (78.7%) had a probability of being avoidable of less than 10% (eFigure in the Supplement).

Only 51 cases of unplanned readmissions (47.2%) presented perfect agreement between the 4 independent reviewers. Among these 51 cases, only 1 was classified as avoidable. The latent class model was also used to assess the individual performance of the 9 reviewers (Table 2). Mean sensitivity was 0.67 (range, 0.20-0.99) and mean specificity was 0.84 (range, 0.49-0.99).

**Table 2.** Performance of the Reviewers

Expert	Specialty	Appraisals, No.	Sensitivity (95%CI)	Specificity (95%CI)	Youden index
1	Epidemiologist	47	0.78 (0.66-0.87)	0.49 (0.34-0.63)	0.27
2	Epidemiologist	52	0.20 (0.08-0.42)	0.94 (0.84-0.98)	0.14
3	Epidemiologist	42	0.72 (0.46-0.88)	0.91 (0.78-0.97)	0.63
4	Infectiologist	53	0.21 (0.03-0.60)	0.99 (0.92-1.00)	0.20
5	Infectiologist	46	0.99 (0.59-1.00)	0.76 (0.64-0.91)	0.75
6	Infectiologist	40	0.49 (0.12-0.88)	0.99 (0.90-1.00)	0.48
7	Pulmonologist	56	0.99 (0.63-1.00)	0.88 (0.75-0.95)	0.87
8	Pulmonologist	47	0.99 (0.48-1.00)	0.85 (0.71-0.95)	0.84
9	Pulmonologist	49	0.69 (0.53-0.81)	0.74 (0.59-0.85)	0.43

### Primary Reason for Readmission

The main causes readmission were relapse or aggravation of a previously known affection (40.1%) and unforeseen readmission for a new affection (24.5%) (Table 3). For readmissions assessed as avoidable, the main reason was a situation involving discharge with a missing or erroneous diagnosis or therapy (19 readmissions [31.7%]). Premature discharge (11 of 60 avoidable readmissions [18.3%] vs 10 of 372 nonavoidable readmissions [2.7%]; *P* < .001) and discharge with missing or erroneous diagnosis or therapy (19 avoidable readmissions [31.7%] vs [5.7%]; *P* < .001) were associated with avoidability.

**Table 3.** Primary Reason Rated by Reviewers

Primary reason	Total reviews (n = 432)	Readmissions, No. (%)		*P* values
		Avoidable (n = 60)	Nonavoidable (n = 372)	
Unforeseen readmission for a new affection	106 (24.5)	1 (1.7)	105 (28.2)	<.001
Complication of surgical care	0	0	0	NA
Complication of nonsurgical care	8 (1.9)	2 (3.3)	6 (1.6)	.31
Drug-related adverse event	23 (5.3)	4 (6.7)	19 (5.1)	.54
Premature discharge	21 (4.9)	11 (18.3)	10 (2.7)	<.001
Discharge with a missing or erroneous diagnosis or therapy	39 (9.0)	19 (31.7)	20 (5.4)	<.001
Other inadequate discharge	27 (6.3)	2 (3.3)	25 (6.7)	.41
Failure of postdischarge follow-up care	21 (4.9)	2 (3.3)	19 (5.1)	.75
Inadequate patient behavior	6 (1.4)	1 (1.7)	5 (1.3)	.60
Relapse or aggravation of a previously known affection	173 (40.1)	18 (30.0)	155 (41.7)	.09
Social readmission	8 (1.9)	0	8 (2.2)	.61

### Factors Associated With Avoidability

The median (IQR) delay between the index hospitalization discharge and the readmission was notably shorter when the readmission was deemed avoidable (4 [6-21] days vs 12 [2-18] days, *P* = .02) (Table 4). None of the other factors related to preadmission, functional and cognitive status, and postdischarge were found as significantly associated with avoidability.

**Table 4.** Unadjusted Comparison of Factors Associated With Readmission

Characteristics	Readmission, No. (%)		*P* value
	Avoidable (n = 15)	Nonavoidable (n = 93)	
Sex			
Men	10 (67.7)	54 (58.1)	.53
Women	5 (33.3)	39 (41.9)	
Age, median (IQR), y	81 (61-92)	77 (65-86)	.43
Cognitive status			
Dementia or Alzheimer disease	1 (7)	9 (9)	>.99
Psychiatric illness	2 (13)	16 (17)	>.99
Frailty	2 (13)	15 (16)	>.99
Admission within the previous year			
Hospitalization	5 (33)	55 (59)	.06
ED visit	2 (13)	22 (24)	.37
Preadmission residence			
Private residence	12 (80)	76 (83)	.81
Nursing home	3 (20)	13 (14)	
Other	0	3 (3)	
Index stay			
PSI class IV-V	11 (73.3)	61 (65.6)	.56
Charlson Comorbidity Index score >0	11 (73.3)	79 (85.0)	.26
Length of index stay, median (IQR), d	3 (1-13)	10 (7-14)	.06
Katz ADL Limitation			
Personal hygiene	3 (20)	28 (30)	.55
Dressing	3 (20)	24 (26)	.76
Toileting	3 (20)	23 (25)	.76
Ambulating	3 (20)	21 (23)	.86
Continence	2 (13)	20 (22)	.73
Feeding	1 (7)	15 (16)	.46
No. of medication at discharge, median (IQR)	7 (2-10)	9 (5-11)	.15
Clinical instability on discharge			
Temperature >37.8 °C	2 (13.3)	5 (5.4)	.25
Heart rate >100 bpm	6 (40.0)	19 (20.4)	.11
Respiratory rate >24/min	1 (6.7)	2 (2.2)	.37
SBP <90 mm Hg	2 (13.3)	5 (5.4)	.25
Unstable oxygenation^a^	0	8 (8.6)	.60
Inability to maintain oral intake	1 (6.7)	4 (4.3)	.53
Altered mental status	1 (6.7)	6 (6.5)	>.99
Postdischarge supports			
Urinary device	0	3 (3)	>.99
Peripheral venous catheter	0	0	NA
Central venous catheter	1 (7)	1 (1)	.26
Tracheostomy	0	1 (1)	>.99
Visiting			
Nurse services	4 (27)	22 (24)	.75
Social services	3 (20)	13 (14)	.69
No. of medication at discharge, median (IQR)	7 (2-10)	9 (5-11)	.15
Readmission			
Readmission through emergency department	12 (80.0)	62 (66.7)	.38
Readmission delay, median (IQR)	4 (6-21)	12 (2-18)	.02

Abbreviations: ED, emergency department; PSI, pneumonia severity index; SBP, systolic blood pressure.

^a^ Hypoxemia was defined as an oxygen saturation rate lower than 90% without supplemental oxygen or lower than 95% with supplemental oxygen or a Pao_2_ lower than 60 mm Hg. Patients who used home oxygen therapy prior to admission were not considered to have unstable oxygenation on discharge.

## Discussion

Our multicenter cohort study showed that only a small proportion of hospital readmissions following hospitalization for pneumonia were avoidable. Less than one-fifth of all unplanned readmissions and less than one-tenth of all readmissions, respectively, were deemed to be avoidable. Furthermore, readmissions deemed avoidable occurred much earlier than those considered nonavoidable.

There has long been discomfort around the use of 30-day readmission rates derived from administrative data, because it is widely known that such automatic indicators do not account for the complexity of clinical situations and thus represent an incomplete capture of low quality of care at the hospital level.^3,4,7,20,21,22^ The low rate of avoidable readmissions that we found contradicts the logic of the HRRP program, which is based upon the notion that they should be mostly avoidable. By contrast, our main finding is in line with other studies investigating the rate of avoidable readmissions for pneumonia, which ranged from 6.3% to 12.5%.^12,13,14,15,16,23^ Nevertheless, these studies were characterized by heterogeneous approaches to defining key terms, data collection, and data analysis.

The novelty of our study consisted of the use of multiple and independent medical record review, and of latent class analysis that was applied to readmissions of patients with pneumonia. The need to apply this method was illustrated by the difficulty for different experts to come to a concordant conclusion regarding avoidable readmission. Even with detailed and exhaustive clinical information, judgements about avoidability varied among reviewers (ie, sensitivity and specificity). A large number of complex factors can potentially influence readmission to the hospital.^15,16,24,25,26,27,28,29,30,31^ Clinical cases of pneumonia are complex, involving a majority of older patients with comorbidities. In some cases, patients are facing difficult social situations, which are known to affect discharge and follow-up. While these considerations remain hypothetical, they show that an algorithm based solely on medico-administrative data cannot be relevant enough to assess whether readmission is avoidable or not.

A secondary objective was to identify factors associated with avoidable readmissions. The difference in time from hospital discharge to readmission was significantly lower in the avoidable readmission group. This finding corroborates doubts about the pertinence of the 30-day readmission interval used by the HRRP program. In 2016, Chin and colleagues^32^ reported that the hospital-level quality signal captured in pneumonia readmission risk was highest between the first and the seventh day postdischarge. Shorr et al^31^ suggested that a longer time interval found a majority of readmissions related to preexistent comorbidity relapse, the patient’s social situation, and residence in nursing home. In our study, we likewise found that a majority of nonavoidable readmissions were caused by a relapse or aggravation of a previously known affection, and that it concerned only a small proportion of avoidable readmissions.

Adverse event detection and measurement is inherently difficult and there is no failsafe method to capture events.^33^ Given the use of 30-day readmission as an indicator for financial penalties and public reporting, our study suggests that the HRRP indicators may unfairly penalize hospitals financially and lower their reputation. The weakness we report here should be considered by health organizations in light of the other previously reported flaws of the 30-day readmission indicators. Several choices are available to policy makers. A first approach would be to avoid reporting of quality using this indicator, which is far from ideal given the limited number of quality-of-care indicators available to health systems. A second would be to structure national reporting systems around the general approach used in this study, ie, a consensus-based expert review of readmissions. However, reviewing manual records is laborious, and the need for multiple and independent experts raises questions about feasibility and opportunity costs.^34^ A third option would be to develop a risk prediction model designed to identify avoidable 30-day readmission of patients hospitalized with pneumonia. However, in order to report a high number of avoidable rehospitalizations and to attain power sufficient to identify factors associated with avoidability, it would be necessary to conduct the same type of study on a larger number of hospitals. While this option remains possible, it would also require more detailed clinical information than is available in administrative claims-based data.^35^ That said, the generalization of digital health records at the hospital level and the development of artificial intelligence algorithms applied to electronic medical records could create a pathway to automated clinical prediction models in the near future.^36,37^

### Limitations

The present study has several important limitations. First, we included patients using administrative claims database specific to the 2 index hospitals and we were unable to capture readmissions in other hospitals. Burke et al^38^ estimated that 20% of 30-day readmissions occur at nonindex hospitals, and that nonindex readmissions had slightly different characteristics in comparison with index readmissions. Therefore, our avoidability analysis might be different if applied to nonindex readmission. Second, we did not manage to identify clinical factors associated with readmissions deemed avoidable, with the exception of time to readmission. Nevertheless, this limitation should be considered in light of our primary outcome regarding the low proportion of avoidable readmissions. We did not expect to observe such a low proportion, which limited the power of the analyses planned in the original protocol. In addition, the low proportion did not make possible the multivariate analysis of time to readmission found to be significantly associated with avoidability in univariate analysis. We cannot therefore exclude that this association was not due to confounding factors, such as patient severity or other characteristics related to patient management. Third, although conducted in 2 large hospitals, our study was limited to a single region of France, and our results might not be fully representative in other contexts and settings.

## Conclusions

In conclusion, our study found that only a small proportion of readmissions following hospitalization for pneumonia were avoidable. Given the complexity of clinical situations, detailed clinical data and multiple independent reviews are essential to judge whether a pneumonia readmission is avoidable. The use of 30-day readmission following a hospitalization for CAP for determining shortcut payments and public reporting may unfairly penalize hospitals.

## Notes

Supplement. **eTable 1.** *ICD-10* Codes That Define Pneumonia **eTable 2.** Description of Scales Used by Physician Reviewers to Rate Readmission Causation and Avoidability **eFigure.** Distribution of Avoidable Probability
