# Supplementary material for: Incidence of Avoidable 30-Day Readmissions Following Hospitalization for Community-Acquired Pneumonia in France
Source: JAMA Netw Open. 2022 Apr 8;5(4):e226574. doi: 10.1001/jamanetworkopen.2022.6574 (PMC8994128; doi:10.1001/jamanetworkopen.2022.6574)
Supplement: Supplement. — eTable 1. ICD-10 Codes That Define Pneumonia eTable 2. Description of Scales Used by Physician Reviewers to Rate Readmission Causation and Avoidability eFigure. Distribution of Avoidable Probability [file jamanetwopen-e226574-s001.pdf]

Supplemental Online Content

Boussat B, Cazzorla F, Le Marechal M, et al. Incidence of avoidable 30-day readmissions following hospitalization for community-acquired pneumonia in France. *JAMA Netw Open.* 2022;5(4):e226574. doi:10.1001/jamanetworkopen.2022.6574

eTable 1. *ICD-10* Codes That Define Pneumonia

eTable 2. Description of Scales Used by Physician Reviewers to Rate Readmission Causation and Avoidability

eFigure. Distribution of Avoidable Probability

This supplemental material has been provided by the authors to give readers additional information about their work.

© 2022 Boussat B et al. *JAMA Network Open.*

eTable 1. ***ICD-10*** **Codes that Define Pneumonia**

ICD-10	Description
Primary diagnosis code of pneumonia	
B01.2	Varicella pneumonia
B20.6	HIV disease resulting in Pneumocystis jirovecii pneumonia
B25.0	Cytomegaloviral pneumonitis
B59	Pneumocystosis
J10.0	Influenza with pneumonia, seasonal influenza virus identified
J11.0	Influenza with pneumonia, virus not identified
J12.x	Viral pneumonia, not elsewhere classified
J13	Pneumonia due to Streptococcus pneumoniae
J14	Pneumonia due to Haemophilus influenzae
J15.x	Bacterial pneumonia, not elsewhere classified
J16.x	Pneumonia due to other infectious organisms, not elsewhere classified
J17.x	Pneumonia in diseases classified elsewhere
J18.x	Pneumonia, organism unspecified
J69.0	Pneumonitis due to inhalation of food and vomit
Primary diagnosis code of sepsis, respiratory failure, or compatible symptoms with a secondary diagnosis code of pneumonia	
A40.x	Streptococcal sepsis
A41.x	Other sepsis
D65	Disseminated intravascular coagulation (defibrination syndrome)
E86.x	Volume depletion
E87.x	Other disorders of fluid, electrolyte and acid-base balance
J80	Adult respiratory distress syndrome
J81	Pulmonary edema
J85.1	Abscess of lung with pneumonia
J90	Pleural effusion, not elsewhere classified
J91	Pleural effusion in conditions classified elsewhere
J96.x	Respiratory failure, not elsewhere classified
O99.5	Diseases of the respiratory system complicating pregnancy, childbirth and the puerperium
R04.2	Haemoptysis
R06.0	Abnormalities of breathing
R07.1	Chest pain on breathing
R07.2	Precordial pain
R07.3	Other chest pain
R07.4	Chest pain, unspecified
R41.0	Disorientation, unspecified
R50.9	Fever, unspecified
R57.1	Hypovolaemic shock
R57.2	Septic shock
R57.9	Shock, unspecified

© 2022 Boussat B et al. *JAMA Network Open.*

R91	Abnormal findings on diagnostic imaging of lung

© 2022 Boussat B et al. *JAMA Network Open.*

eTable 2. Description of Scales Used by Physician Reviewers to Rate Readmission Causation and Avoidability

RATING	CAUSATION	AVOIDABILITY
1	No evidence for management causation	No evidence for preventability
2	Slight evidence for management causation	Slight evidence for preventability
3	Management causation less than 50-50 but close call	Preventability less than 50-50 but close call
4	Management causation more than 50-50 but close call	Preventability more than 50-50 but close call
5	Strong evidence for management causation	Strong evidence for preventability
6	Virtually certain evidence for management causation	Virtually certain evidence for preventability

For each domain, a rating of 4 or more indicated that the reviewer felt that evidence existed for causation or avoidability in that case.

eFigure 1

Distribution of avoidable probability

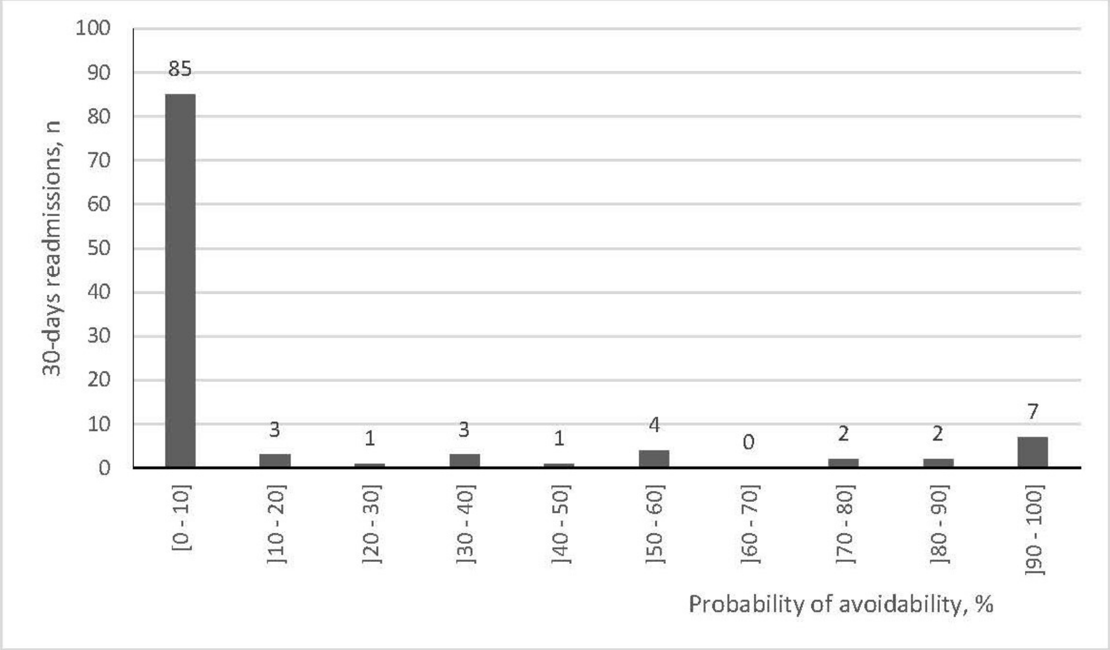

© 2022 Boussat B et al. *JAMA Network Open.*
